# Supplementary material for: Towards greater integration: Prospects for the development of agri-food trade between the EU and RCEP countries
Source: PLoS One. 2025 Jul 21;20(7):e0328866. doi: 10.1371/journal.pone.0328866 (PMC12279148; doi:10.1371/journal.pone.0328866)
Supplement: S1 Appendix — (DOCX) [file pone.0328866.s001.docx]

**S1 Appendix. Full nomenclature of the analyzed product groups**

**1** Live animals; animal products

**2** Meat and edible meat offal

**3** Fish and crustaceans, molluscs and other acquatic invertebrates

**4** Dairy produce; birds' eggs; natural honey; edible products of animal origin, not elsewhere specified or included

**5** Products of animal origin, not elsewhere specified or included

**6** Live trees and other plants; bulbs, roots and the like; cut flowers and ornamental foliage

**7** Edible vegetables and certain roots and tubers

**8** Edible fruit and nuts; peel of citrus fruit or melons

**9** Coffee, tea, mate and spices

**10** Cereals

**11** Products of the milling industry; malt; starches; inulin; wheat gluten

**12** Oil seeds and oleaginous fruits; miscellaneous grains, seeds and fruit; industrial or medicinal plants; straw and fodder

**13** Lac; gums, resins and other vegetable saps and extracts

**14** Vegetable plaiting materials; vegetable products not elsewhere specified or included

**15** Animal or vegetable fats and oils and their cleavage products; prepared edible fats; animal or vegetable waxes

**16** Preparations of meat, of fish or of crustaceans, molluscs or other aquatic invertebrates

**17** Sugars and sugar confectionery

**18** Cocoa and cocoa preparations

**19** Preparations of cereals, flour, starch or milk; pastrycooks' products

**20** Preparations of vegetables, fruit, nuts or other parts of plants

**21** Miscellaneous edible preparations

**22** Beverages, spirits and vinegar

**23** Residues and waste from the food industries; prepared animal fodder

**24** Tobacco and manufactured tobacco substitutes
