# Supplementary material for: Towards greater integration: Prospects for the development of agri-food trade between the EU and RCEP countries
Source: PLoS One. 2025 Jul 21;20(7):e0328866. doi: 10.1371/journal.pone.0328866 (PMC12279148; doi:10.1371/journal.pone.0328866)
Supplement: S1 Fig — (DOCX) [file pone.0328866.s002.docx]

S1 Figure. Value of agri-food trade between the EU and RCEP in 2013-2022 (billions EUR)

Source: Comext-Eurostat data.
