# Supplementary material for: Towards greater integration: Prospects for the development of agri-food trade between the EU and RCEP countries
Source: PLoS One. 2025 Jul 21;20(7):e0328866. doi: 10.1371/journal.pone.0328866 (PMC12279148; doi:10.1371/journal.pone.0328866)
Supplement: S1 Table — (DOCX) [file pone.0328866.s003.docx]

S1 Table. Descriptive statistics for intra-industry trade

| **HS** | **MIN** | | | | **MAX** | | | | **Average** | | | | **Coefficient of variation (%)** | | | |
| --- | --- | --- | --- | --- | --- | --- | --- | --- | --- | --- | --- | --- | --- | --- | --- | --- |
|  | IIT | HIIT | VIITlow | VIIThigh | IIT | HIIT | VIITlow | VIIThigh | IIT | HIIT | VIITlow | VIIThigh | IIT | HIIT | VIITlow | VIIThigh |
| 1 | 0.23 | 0.00 | 0.03 | 0.01 | 0.39 | 0.08 | 0.38 | 0.16 | 0.28 | 0.03 | 0.16 | 0.08 | 19.97 | 90.22 | 71.22 | 70.77 |
| 2 | 0.15 | 0.03 | 0.03 | 0.00 | 0.43 | 0.18 | 0.18 | 0.07 | 0.24 | 0.08 | 0.11 | 0.04 | 42.62 | 67.43 | 47.18 | 66.76 |
| 3 | 0.24 | 0.01 | 0.10 | 0.13 | 0.38 | 0.07 | 0.15 | 0.18 | 0.33 | 0.05 | 0.14 | 0.14 | 14.62 | 46.97 | 15.37 | 14.23 |
| 4 | 0.07 | 0.00 | 0.00 | 0.04 | 0.19 | 0.02 | 0.01 | 0.18 | 0.12 | 0.01 | 0.01 | 0.10 | 39.17 | 90.26 | 30.10 | 49.90 |
| 5 | 0.70 | 0.00 | 0.52 | 0.14 | 0.79 | 0.01 | 0.65 | 0.19 | 0.73 | 0.00 | 0.56 | 0.17 | 4.42 | 144.28 | 8.61 | 10.50 |
| 6 | 0.54 | 0.00 | 0.03 | 0.39 | 0.60 | 0.05 | 0.14 | 0.52 | 0.56 | 0.02 | 0.09 | 0.45 | 3.97 | 76.67 | 41.87 | 9.51 |
| 7 | 0.37 | 0.11 | 0.09 | 0.13 | 0.51 | 0.18 | 0.16 | 0.23 | 0.44 | 0.14 | 0.12 | 0.18 | 10.93 | 19.32 | 24.23 | 19.83 |
| 8 | 0.19 | 0.00 | 0.02 | 0.11 | 0.29 | 0.06 | 0.06 | 0.22 | 0.23 | 0.02 | 0.05 | 0.15 | 15.59 | 105.07 | 29.11 | 22.80 |
| 9 | 0.08 | 0.00 | 0.00 | 0.07 | 0.25 | 0.02 | 0.01 | 0.24 | 0.19 | 0.01 | 0.01 | 0.17 | 30.34 | 63.88 | 37.53 | 34.40 |
| 10 | 0.02 | 0.00 | 0.01 | 0.01 | 0.07 | 0.01 | 0.04 | 0.04 | 0.05 | 0.00 | 0.02 | 0.02 | 35.10 | 125.17 | 46.14 | 50.81 |
| 11 | 0.13 | 0.01 | 0.02 | 0.07 | 0.21 | 0.05 | 0.08 | 0.12 | 0.16 | 0.03 | 0.03 | 0.10 | 16.40 | 55.49 | 80.96 | 19.07 |
| 12 | 0.29 | 0.01 | 0.10 | 0.05 | 0.53 | 0.16 | 0.46 | 0.07 | 0.39 | 0.09 | 0.24 | 0.06 | 20.99 | 73.12 | 58.54 | 17.05 |
| 13 | 0.45 | 0.01 | 0.00 | 0.09 | 0.75 | 0.41 | 0.26 | 0.74 | 0.63 | 0.17 | 0.08 | 0.38 | 19.77 | 103.10 | 130.16 | 67.07 |
| 14 | 0.02 | 0.00 | 0.00 | 0.00 | 0.05 | 0.00 | 0.04 | 0.05 | 0.04 | 0.00 | 0.03 | 0.01 | 21.48 | 197.28 | 55.28 | 150.79 |
| 15 | 0.11 | 0.01 | 0.01 | 0.03 | 0.23 | 0.08 | 0.06 | 0.21 | 0.17 | 0.04 | 0.03 | 0.11 | 27.33 | 83.71 | 61.99 | 78.15 |
| 16 | 0.19 | 0.01 | 0.01 | 0.10 | 0.26 | 0.08 | 0.12 | 0.18 | 0.23 | 0.03 | 0.06 | 0.14 | 12.70 | 87.72 | 81.70 | 23.19 |
| 17 | 0.31 | 0.00 | 0.17 | 0.06 | 0.49 | 0.11 | 0.33 | 0.21 | 0.41 | 0.05 | 0.23 | 0.13 | 14.92 | 73.78 | 25.47 | 39.15 |
| 18 | 0.09 | 0.01 | 0.00 | 0.04 | 0.52 | 0.41 | 0.02 | 0.40 | 0.36 | 0.17 | 0.01 | 0.18 | 40.25 | 107.86 | 49.32 | 81.97 |
| 19 | 0.22 | 0.01 | 0.05 | 0.05 | 0.33 | 0.05 | 0.15 | 0.22 | 0.28 | 0.03 | 0.10 | 0.16 | 13.31 | 54.73 | 40.66 | 36.27 |
| 20 | 0.38 | 0.08 | 0.03 | 0.19 | 0.60 | 0.25 | 0.14 | 0.36 | 0.52 | 0.14 | 0.09 | 0.29 | 14.95 | 46.74 | 45.04 | 21.23 |
| 21 | 0.53 | 0.00 | 0.00 | 0.48 | 0.62 | 0.10 | 0.04 | 0.59 | 0.60 | 0.03 | 0.02 | 0.54 | 5.93 | 118.84 | 52.36 | 7.88 |
| 22 | 0.15 | 0.01 | 0.03 | 0.07 | 0.18 | 0.04 | 0.04 | 0.12 | 0.16 | 0.02 | 0.04 | 0.10 | 7.82 | 35.32 | 7.39 | 18.70 |
| 23 | 0.35 | 0.03 | 0.26 | 0.03 | 0.65 | 0.17 | 0.45 | 0.20 | 0.51 | 0.08 | 0.36 | 0.07 | 23.87 | 59.89 | 20.41 | 85.48 |
| 24 | 0.28 | 0.00 | 0.08 | 0.20 | 0.66 | 0.02 | 0.19 | 0.47 | 0.43 | 0.00 | 0.12 | 0.31 | 33.95 | 162.92 | 34.48 | 32.97 |

Source: own calculations based on Comext-Eurostat data
