# Supplementary material for: Towards greater integration: Prospects for the development of agri-food trade between the EU and RCEP countries
Source: PLoS One. 2025 Jul 21;20(7):e0328866. doi: 10.1371/journal.pone.0328866 (PMC12279148; doi:10.1371/journal.pone.0328866)
Supplement: Attachment — Submitted filename: Rebuttal letter.docx [file pone.0328866.s004.docx]

We would like to thank the Reviewers for the feedback and all the comments that helped us improve the paper. Below we explain the changes made in line with the reviewer’s recommendations. We believe that the revisions improved the quality and scientific soundness of the paper. All additions can be seen in the file with tracked changes.

Reviewer #1

Recommendation:

1. I suggest that under table 1 you put full nomenclature of the analyzed product groups 1-24).

According to your suggestion, a full nomenclature of the analyzed product groups was inserted under Table 1.

Reviewer #2

Recommendations:

1. Incorporate more recent literature and diverse perspectives, particularly on recent shifts in EU-RCEP relations due to global trade policy changes.

We incorporated a more specific description of EU challenges due to global trade policy changes, particularly focusing on Asia-Pacific relations based on recent literature. An appropriate part covering the EU strategy to pursue FTAs with several Indo-Pacific nations as a counterbalance to RCEP's influence was added to the introduction. Moreover, risks for the EU posed by the US-China trade war were articulated, and potential opportunities for the EU to leverage its partnerships in Asia were mentioned.

2. Add graphs or charts in the Results section to visually convey trends and make it easier to interpret the longitudinal changes in trade indices.

The trend lines were added to Figure 1 (results of ESI and PSI). Moreover, an additional figure was added to better visualize changes in intra-industry trade.

3. Clarify the advantages of using highly disaggregated data in the methodology section, explaining its impact on the accuracy and relevance of findings.

According to your suggestion, we have developed the validity of using disaggregated data. Previously, information about this was included in two places in the methodology:

“We aimed for the highest possible level of disaggregation as it significantly impacts the values of the indicators we used [31]. As the level of data disaggregation increases, their values tend to decrease, giving more accurate estimation [32].”

“Using highly disaggregated data can help conduct an in-depth analysis and obtain more information on the import and export patterns of the countries, which allows a better understanding of trade dynamics [35].”

We also added an additional explanation:

“Disaggregated data enables the study to identify trade patterns specific to individual product categories, leading to more accurate measurements of trade similarity and intra-industry trade levels. By tracking exports and imports at the 6- and 8-digit HS code level, the analysis can capture variations across a broad range of agri-food products, enhancing the specificity and relevance of the results [36].”

4. Make specific recommendations for policymakers and industry stakeholders, linking findings directly to practical applications.

We created a more detailed recommendation, suggesting specific actions and their effects, with the goal of increasing trade between the EU and RCEP.

5. Discuss potential limitations of the study, such as data availability or specific trade barriers, and suggest avenues for further research.

In the description, we added limitations to our study, where we focused on non-tariff barriers to trade between EU and RCEP countries. In addition, we described in more detail the possibilities for future research.

6. Include a more detailed examination of non-tariff barriers affecting agri-food trade between the EU and RCEP, such as regulatory and quality standards.

In line with your recommendation, in the results and discussion section, we paid more attention to non-tariff barriers affecting trade in agri-food products between the EU and RCEP.

Reviewer #3

Recommendations:

Abstract

1. The research contributions of the paper should be articulated more clearly. The abstract is not representative of the content and contributions of the paper. The abstract does not seem to properly convey the rigor of research.

We have revised the abstract to clearly articulate the paper’s contributions. The updated abstract now emphasizes the study’s primary focus on identifying opportunities for sectoral cooperation and competitive dynamics within EU-RCEP agri-food trade, addressing an acknowledged gap in existing research. By specifically highlighting findings such as stable but moderate competition and the potential for EU exports of high-quality agri-food products, we aim to showcase both the significance and the depth of our analysis. The revised abstract provides a clearer representation of the paper's objectives, contributions, and results.

2. Aside from the aim stated in the title, the research gap and the goals of the research are not specified which leads to the reader missing the significance of the research.

In the revised abstract, we explicitly outline the research gap - the lack of detailed analysis of EU-RCEP agri-food trade relations. The revised version now states that the goal of the study is to assess sectoral cooperation opportunities and competitive dynamics, establishing a foundation for potential future trade negotiations. These additions underscore the study's purpose and relevance, allowing readers to understand better the strategic significance of our research in shaping EU-RCEP trade policy.

Introduction

1. The introduction section is detailed, but needs a significant amount of reorganization. It could be strengthened by adding more recent references.

More recent references have been added, as suggested. After these changes, the introduction uses 21 literature sources, of which more than half were published in the last 5 years and 8 of them in the last 3 years. Moreover, the introduction was reorganized by adding a paragraph on recent policy shifts affecting EU-RCEP relations. Furthermore, more emphasis was placed on the novelty of the study.

2. Please add as sentence or two to clearly recap how your study differs from what has already been done in literature to ascertain the contributions more strongly

According to this suggestion, we added the following sentences: “The research so far is concerned solely with macro-level relations between two groupings or relations between the EU and single countries in the Asia-Pacific region. Sectoral-level studies, to date, are of a descriptive nature or covering only a few groups of products”.

3. More explanation is needed for where there is a research gap and what the goals of the research are. The research gap and the goals of the research are not explained in detail which leads to the reader missing the significance of the research.

According to this suggestion, we added the following sentences: “the novelty of our research lies in a detailed analysis of agri-food trade between the EU and RCEP, covering 24 chapters of agri-food products.”, and “we aim to analyse trade potential for each product category between the EU and RCEP based on empirical data. Therefore extending the current state of knowledge through evidence-based insides”.

4. The research idea should be linked to multiple problems the research is trying to address so that the findings have relevance.

This issue was solved by adding a paragraph-length description of recent shifts in trade policies and their link to the EU-RCEP situation. We mentioned EU strategy to pursue FTAs with several Indo-Pacific nations as a counterbalance to RCEP's influence. Moreover, risks for the EU posed by the US-China trade war were articulated, and potential opportunities for the EU to leverage its partnerships in Asia were mentioned. “For the EU, these developments pose a dual challenge: leveraging its bilateral agreements with RCEP members to secure market access while navigating the effects of large-scale initiatives of the US and China”. The issues mentioned correspond directly with our research idea to “map the baseline situation for potential negotiations of FTA between the EU and RCEP regarding agri-food products”.

Literature Review

1. Sources are out of date. More recent studies (2021-2024) should be included. Also, it should lead up to the research questions in a logical manner.

The literature review has been extended to include more recent literature on the subject. The novelty of the study and theory-based justification for the analyses performed were also emphasized. As a result, the research gap is better addressed, and the study’s contributions are clarified in more detail.

2. Please discuss learning theories and tie them to both, the research gap addressed by the paper as well as to the factors in the research model.

Thank you for this comment. Now, the literature review section discusses theories that serve as a background for the analyses performed. The theory of comparative advantage, the theory of overlapping demand, the theory of intra-industry trade, and the customs union theory were considered to address the research gap and show the study’s importance.

3. The literature review is insufficient in its addressal of the research gap and research model.

In line with the reviewer’s recommendation, the literature review has been extended to clarify the research gap and the study’s contributions in more detail. We took advantage of more recent studies and employed a few theories, which constitute the foundations for the analyses performed.

Methodology

1. The methodology section needs more details and a drastic revision.

We introduced more details in the methodology. We have added information about the rationale for using disaggregated data, and we have also focused on how the chosen methods fit our research questions. In addition, we divided the methodology into sections.

2. I suggest using regression analysis.

We agree that such an addition could be beneficial. However, we treat it as an idea for different studies, as it is not crucial for fulfilling our goal. We included the idea of using regression analysis as a future direction of research. A regression analysis or gravity model can definitely help assess the strength of possible effects (trade shifting and creation) on trade between the EU and RCEP.

3. The subsections of participants, instruments, data collection procedures, and data analysis should be separately given.

According to this remark, we restructured this section, splitting it into “Data Collection” and “Calculation Procedure”, to boost readability.

4. The items in the instrument used, demographic information, reliability and validity information, any statistical or data analysis should be presented in detail.

Additional information has been added to the Materials and Methods section. We focused on further clarifying the advantages of using highly disaggregated data and explaining its impact on the accuracy and relevance of results. Moreover, we explicitly gave the number of countries considered in the analysis. Furthermore, the number of commodities analyzed under the 8-digit HS classification was provided.

5. The reason for using specific analysis is not clearly mentioned. Justification for using a specific methodology or instrument will make it more understandable. Adding more details in this section can give more clarity to the readers.

In line with your recommendation, we have added more details about the methods, as well as a justification for choosing a particular method of analysis.

6. The methodology used should be justified in the article in the light of the research questions (i.e. why is the chosen methodology the best approach to answer the research questions).

In the data and methods section, we have also added an explanation of how the chosen methods will answer the research questions.

Discussion

1. Improve the discussion section to better ascertain what is unique / novel about your findings.

In accordance with your recommendation, both in the introduction and in the discussion, we drew attention to the novelty of our article.

2. Explain in detail how the article contributes to new knowledge in the domain.

We have added an explanation of how our study creates new knowledge in the domain.

3. The manuscript lacks policy recommendations.

We created a more detailed recommendation, suggesting specific actions and their effects, with the goal of increasing trade between the EU and RCEP.

Conclusion

1. Update the conclusion to include the newly formulated theoretical contributions.

Following your recommendation, we have formulated what contribution our article makes to theory. The following sentence was added: “This study contributes to the theoretical discourse on regional trade integration by illustrating how structural trade differences in agri-food products affect intra-industry trade, by empirically linking quality differentiation (e.g., VIIThigh and VIITlow) to trade potential.”

2. Mention the limitations of the study and prospects for future research.

In the description, we added limitations to our study, where we focused on non-tariff barriers to trade between EU and RCEP countries. In addition, we described in more detail the possibilities for future research. The following sentences were added “. Conversely, a limitation of our study is the failure to measure a specific effect of non-tariff barriers, such as sanitary and phytosanitary regulations and environmental regulations. Restrictive EU standards for food quality, health protection, and CO₂ emissions do not always align with those of RCEP countries, resulting in the need for additional certification and product adjustments, which can hinder trade.” “Future studies should also consider non-tariff barriers and their impact on trade. For this purpose, it would be possible to use regression analysis or gravity model.”

3. Summarize the key results in a compact form and re-emphasize their significance.

We have added key findings to the summary: “Therefore, key findings include, firstly, moderate competition in agri-food trade between the EU and RCEP, and secondly, the dominance of high-quality vertical trade flows (VIIThigh) suggests the EU's competitive advantage in high-value agri-food products. We also observed weak but strengthening intra-industry trade in select product groups, which highlights untapped potential for trade cooperation. These insights underscore the need for harmonized strategic export policies to develop trade relations between EU and RCEP countries.”

4. Summarize how the article contributes to new knowledge in the domain.

In the conclusions, we added what new knowledge the article brings to the domain. We focused mainly on structural differences that affect intra-industry trade and differences in product quality that affect the potential for trade development.
