# Supplementary material for: Towards greater integration: Prospects for the development of agri-food trade between the EU and RCEP countries
Source: PLoS One. 2025 Jul 21;20(7):e0328866. doi: 10.1371/journal.pone.0328866 (PMC12279148; doi:10.1371/journal.pone.0328866)
Supplement: Attachment — Submitted filename: Rebuttal letter_2.docx [file pone.0328866.s005.docx]

We would like to thank the Reviewer for the feedback and all the comments that helped us improve the paper. Below we explain the changes made in line with the reviewer’s recommendations. We believe that the revisions improved the quality and scientific soundness of the paper. All additions can be seen in the file with tracked changes.

Recommendations:

Major comment 1 – issues with Table 1 and the main analysis

1a. The methodology description is insufficient. Who is the exporter and who is the importer? Since it matters for saying whether the trade is high vertical or low vertical. From the discussion RCEP appears to be the importer, but that should be made explicit in the formulas (e.g. equation 6) and in the table heading.

We agree that it was not explicitly stated when describing the methodology that we are analysing intra-industry trade from an EU perspective, so when describing the indicators for horizontal and vertical trade, we have indicated that we are referring to the EU.
We have also made a change to the name of the table.

1b. How did you collapse this from CN8 to HS2 headings? Probably you took the average, but essential things like this should be explained for reproducibility.

A detailed explanation has been added to the Materials and Methods section: “In the analysis of intra-industry trade, the CN classification at the 8-digit level was used, which is a more detailed extension of the HS classification. This allows each CN code to be assigned to the appropriate product group at the 2-digit HS level. Ratios of unit values of exports (UVX) to imports (UVM) are calculated at the level of 8-digit CN codes. Based on the results for individual CN codes, the value of products traded within horizontal and vertical trade can then be determined for each product group classified at the 2-digit HS level. This approach makes it possible to obtain aggregated indicators for each general product group at the 2-digit HS level. In other words, the analysis reveals the value of products within a given group (2-digit HS) that constitutes a specific type of intra-industry trade, based on classifications made at the detailed CN level”*.*

1c. In the conclusion you write “high-quality vertical trade was observed in most product groups” - This statement may be true but requires more explicit analysis. Not even a percentage of HS2/HS6 headings for which this is the case is provided in the text.

In both the description of the results and the summary, a clarification was added as to the share of high-quality products in intra-industry trade between EU and RCEP countries.

Major comment 2

Given the repeated references to EU exports of high-quality goods, one would have expected literature on Geographical Indications (GI) to be cited. These protected regional foods have been shown to have higher unit values, and their protection plays a key role in trade negotiations. By asking trading partners to protect them, the EU hopes/aims to export even more of its high-quality products, as imitations are taken off the market. The abstract has the sentence “While intra-industry trade remains limited due to geographic distance, there is notable potential for expanding high-quality EU agri-food exports” – the legal protection of GIs in RCEP countries (to the extend they are not already subject to a bilateral FTA like e.g. Japan is) would be a channel for that.

Thank you for this particularly useful remark! A paragraph length explanation with literature on Geographical Indications has been added to the discussion, linking our findings with other studies and already existing GI provisions between the EU and RCEP countries.

Minor comments

Line 97-98: Did you check relevant policy documents, e.g. the EU Impact Assessment of the EU-Indonesia FTA available at https://policy.trade.ec.europa.eu/analysis-and-assessment/sustainability-impact-assessments_en It definitely has relevant findings, e.g. on p. 9 "For agri-foods, the model predicts that the agreement could lead to increased production of dairy and alcoholic beverages in the EU"

Thank you for pointing out this omission. The following explanation has been added to the introduction: “Some relevant policy assessments–Sustainability Impact Assessments–of ongoing negotiations exist [22]; however, these studies are limited to bilateral contexts. We aim to map a comprehensive view of the potential of agri-food trade between the EU and RCEP groupings”. Moreover, the discussion has been enhanced by some provisions of the Sustainability Impact Assessment of the EU–Indonesia Free Trade Agreement.

Line 216 “trade in agri-food products as being one of the most sensitive when negotiating trade liberalization” – indeed, all the more reason to cite literature on GIs. Their protection by trading partners is often seen as counterweight for increased market access into EU market.

This comment has been covered by changes based on previous remarks.

Line 248 “8-digit HS”; technically this is CN8, since the HS is worldwide, but beyond HS6 countries use their own systems (and the EU uses CN8)

Changes have been made and it has been explained what the CN8 classification is in the Materials and Methods section.

Line 262 “c denotes shares of exported goods in the total agri-food export”- you mean x denotes this? And c is the index of goods from 1 to n?

This misunderstanding has been clarify by stating that x indicates shares of exported goods in the total agri-food export, c denotes exported commodity.

Line 275 you need to add “of Intra-industry trade (IIT)” to the Gruber-Lloyd index

A change has been made, as you have suggested.

Line 316 eqn7 - give this a different name than (3), since the formula is different

Following your recommendation, the name of the index has been changed. It is now the Quality-Differentiated IIT index to emphasize that it analyses intra-industry trade by vertical and horizontal trade, taking into account qualitative differences.
